# Expression of Cell Cycle Markers and Proliferation Factors during Human Eye Embryogenesis and Tumorigenesis

**DOI:** 10.3390/ijms23169421

**Published:** 2022-08-20

**Authors:** Josipa Marin Lovrić, Natalija Filipović, Ljubo Znaor, Anita Rančić, Joško Petričević, Nenad Kunac, Violeta Šoljić, Mirna Saraga-Babić, Katarina Vukojević

**Affiliations:** 1 Department of Ophthalmology, University Hospital of Split, 21000 Split, Croatia; 2 Department of Anatomy, Histology and Embryology, University of Split School of Medicine, Šoltanska 2, 21000 Split, Croatia; 3 Department of Ophthalmology, University of Split School of Medicine, Šoltanska 2, 21000 Split, Croatia; 4 Department of Pathology, University of Mostar School of Medicine, 88000 Mostar, Bosnia and Herzegovina; 5 Department of Pathology, University Hospital of Split, 21000 Split, Croatia; 6 Department of Histology and Embryology, University of Mostar School of Medicine, 88000 Mostar, Bosnia and Herzegovina; 7 Faculty of Health Studies, University of Mostar, 88000 Mostar, Bosnia and Herzegovina; 8 Department of Anatomy, University of Mostar School of Medicine, 88000 Mostar, Bosnia and Herzegovina

**Keywords:** eye development, retinoblastoma, choroid melanoma, p19, Ki67, PDL-1, MSX1, MSX2, pRB, CYCLA2

## Abstract

The expression pattern of the markers p19, Ki-67, MSX1, MSX2, PDL1, pRB, and CYCLINA2 was quantitatively and semiquantitatively analyzed in histologic sections of the developing and postnatal human eye at week 8, in retinoblastoma, and in various uveal melanomas post hoc studies by double immunofluorescence. The p19 immunoreactivity characterized retinal and/or choroidal cells in healthy and tumor tissues: expression was lower in the postnatal retina than in the developing retina and retinoblastoma, whereas it was high in epithelioid melanomas. Ki67 expression was high in the developing eye, retinoblastoma, and choroidal melanomas. MSX1 and MSX2 expression was similar in the developing eye and retinoblastoma, whereas it was absent in the postnatal eye. Their different expression was evident between epithelioid and myxoid melanomas. Similarly, PDL1 was absent in epithelioid melanomas, whereas it was highly expressed in developing and tumor tissues. Expression of pRB and CYCA2 was characteristic of developing and tumorous eye samples but not of the healthy postnatal eye. The observed expression differences of the analyzed markers correlate with the origin and stage of cell differentiation of the tissue samples. The fine balance of expression could play a role in both human eye development and ocular tumorigenesis. Therefore, understanding their relationship and interplay could open new avenues for potential therapeutic interventions and a better understanding of the mechanisms underlying the developmental plasticity of the eye and the development of neoplasms.

## 1. Introduction

The human eye develops between the 3rd and 10th week of development from the neuroectoderm in the head region. Among other ocular structures, the neuroectoderm gives rise to the retina and its surrounding mesenchyme, which condenses into a pigmented, highly vascularized layer, the choroid. During embryogenesis, the timing of cell fate determination regulates the different cell types, which is thought to be controlled by a tightly coordinated regulatory program of proliferation, cell cycle termination, and differentiation [1]. Because of the close relationship between developmental plasticity of the eye and tumorigenesis, these regulatory programs might be reactivated during ocular carcinogenesis [2]. However, the understanding of this complex gene regulatory network and the mechanisms underlying ocular carcinogenesis has not been fully elucidated [3]. Retinoblastoma (RB) is the most common primary intraocular carcinoma in children. It arises from the neuroectoderm, presumably primitive retinal stem cells that have the ability to differentiate into multiple retinal cell types, including horizontal, amacrine, Müller cells, and photoreceptors [4]. In contrast, uveal melanoma primarily affects adults and is six times more common than RB. Choroidal melanomas can arise from preexisting choroidal nevi or de novo. Choroidal melanoma most commonly affects the choroid (posterior melanoma) and/or ciliary body [5]. Uveal melanoma can be divided into a spindle cell, mixed cell, or epithelioid cell type [6,7].

Proliferative activity detectable by nuclear Ki-67 expression is synchronised by coordinated expression of regulatory proteins of various morphogens, growth factors, and homeodomain proteins [8]. RB1 encodes a nuclear protein that is ubiquitously expressed and involved in the G1 to S transition (cell cycle regulation). Members of the cyclin-dependent kinase (cdk) system phosphorylate the RB protein prior to entry into S phase. This phosphorylation allows loss of binding activity and release of cellular proteins. On the other hand, pathogenic RB1 variants allow the expression of proteins that have lost their cell cycle regulatory functions [9]. To enable a fine balance in the inhibition of cell growth during organogenesis and also in tumor suppression, the cyclin-dependent kinase (Cdk) inhibitor p19INK4d can induce terminal differentiation of cells by blocking cell cycle progression [8]. Histological differentiation during embryogenesis represents a balance between the proliferation of progenitor cells and their differentiation into mature cells [10]. Ki67 is a nuclear protein that is expressed in all active phases of the cell cycle (G1, S, G2, and mitosis) but is not found in cells that are at rest (G0). This makes it an excellent marker for evaluating and distinguishing proliferating cells and as a diagnostic marker for detecting proliferative activity in neoplastic tissue [11]. On the other hand, cell differentiation allows immature embryonic cells to specialize. Cyclins and cyclin-dependent kinases (Cdk) play an important role in cell cycle control mechanisms, and their activity is positively regulated by phosphorylation and negatively regulated by Cdk inhibitors (CDKI). INK4A inhibits G1 phase, which is mediated by G1 cyclin-dependent kinases 4 and 6, which phosphorylate and inactivate the RB protein, allowing entry into S phase of the cell cycle. The arrest of G1 phase of the cell cycle must be bypassed prior to cell division. Loss of INK4A function prevents INK4A from binding to CDK4 and promotes RB activation through hyperphosphorylation, leading to uncontrolled cell cycle progression. p19INK4d belongs to an INK4-CDKI family and inhibits the activity of Cdk4 and Cdk6, which are responsible for cell transition from G1 to S phase. It is highly expressed in adult tissues, and its loss or insufficient function has been described in various types of neoplasms. [12].

Msh homeobox genes regulate transcriptional repressors that promote epithelial- mesenchymal transformation (EMT) during embryogenesis [13]. Among them, Msx1 and Msx2 (muscle segment homeodomain proteins 1 and 2) are involved in several signalling pathways in the development of different organs, and their biological function depends on the molecular context in which they act [8]. The Msx1 lineage does not contribute to retinogenesis at postnatal stages [13], whereas overexpression of Msx2 blocks cell proliferation and allows terminal cell differentiation [14]. MSX2 expression has been identified as a biomarker for good prognosis in patients with malignant melanoma [15]. One of the new concepts of tumorigenesis is escape from the immune system. An important role in the regulation of the immune system is played by PD-1 receptor and PD-L1 ligand expression. The interaction between PD-1 and PD-L1 leads to inhibited activation of T CD8+ lymphocytes. PD-L1 is constitutively expressed in T cells, B cells, dendritic cells, and mesenchymal stem cells and inducible to a higher extent in both immune cells and a variety of non-immune cells not associated with the cell lineage. In melanomas, strong expression of the PD-1 receptor and its PD-L1 ligand is generally observed. The exact mechanisms leading to the upregulation of PD-L1 expression on the surface of tumor cells are currently the subject of intense research [16].

Cyclins, as binding and activating partners of Cdk, are the key factor of the regulatory components of the cell cycle. Cyclin A2 is a regulator of cell proliferation and as such is frequently found at high levels in human cancers [17]. However, the level of this cyclin does not always correlate with tumor invasiveness. Its involvement in the control of cytoskeletal dynamics and cell movement has revealed its role in cytokinesis and subsequent epithelial to mesenchymal transition (EMT). In addition, each phase of the cell cycle is characterized by distinct complexes between cyclins and Cdks responsible for deactivation of Retinoblastoma protein (Rb). The Rb protein is progressively deactivated by phosphorylation at specific residues, as the hypophosphorylated form of Rb inhibits cell cycle progression and entry into mitosis [18].

Many studies have investigated the proliferation and differentiation of tumors and carcinomas, but not in the context of the close relationship between developmental plasticity of the eye and the occurrence of ocular neoplasms. Therefore, the interplay of cell cycle and proliferation markers in both types of intraocular tumors, retinoblastoma and choroidal melanoma, was studied and compared with their expression in the developing and normal postnatal adult human eye. Understanding the interplay of these markers could contribute to early diagnosis and more effective treatment.

## 2. Results

**Figure 1:**
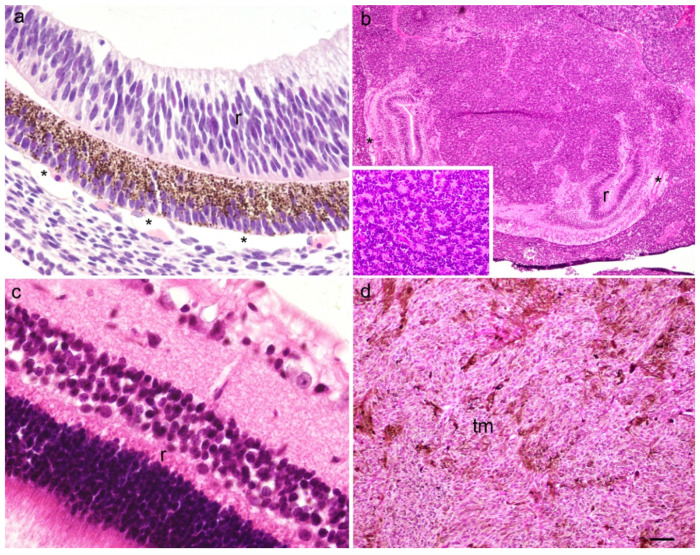
A section through the eye of an 8-week human embryo (**a**), retinoblastoma (**b**), normal human eye (**c**) and myxoid melanoma (**d**); inset on the panel (**b**) present Flexner–Wintersteiner rosette, r—retina, *—choroidea, tm—tumor tissue. Haematoxylin and Eosin staining, scale bar ((**a**,**c**) = 25 μm), ((**b**) = 6 μm), ((**d**) = 12 μm).

**Figure 2:**
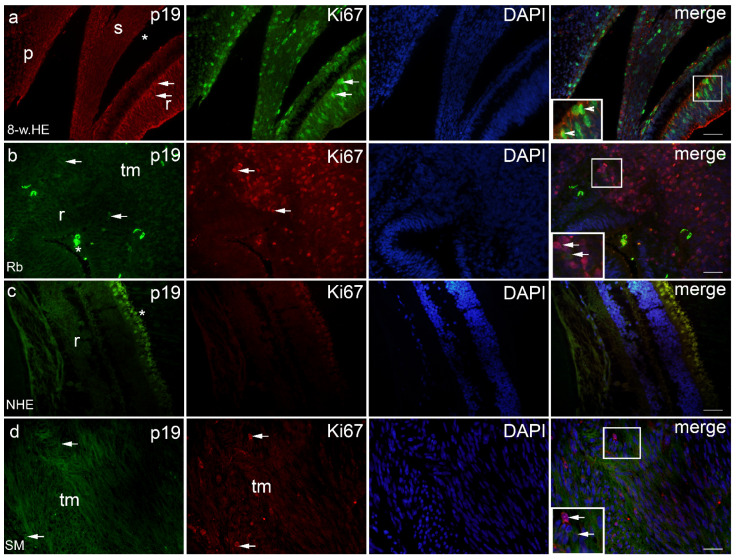
p19 and Ki67 positive cells (arrows) can be seen in the eye section of an 8-week human embryo-8-w.HE (**a**), retinoblastoma–Rb (**b**), normal human eye–NHE (**c**) and spindle melanoma-SM (**d**); r—retina, *—choroidea, tm—tumor tissue, s—sclera, p—palpebra. Co-localization of p19 and Ki67 positive cells can be seen in panel (**a**) (arrowheads). Double immunofluorescence staining to p19 (first column), Ki67 (second column), DAPI (third column), merge (fourth column). Magnification frames provide detail with positive cells (arrows) or co-localization (arrowheads). Scale bar for all panels is 25 μm.

**Figure 3:**
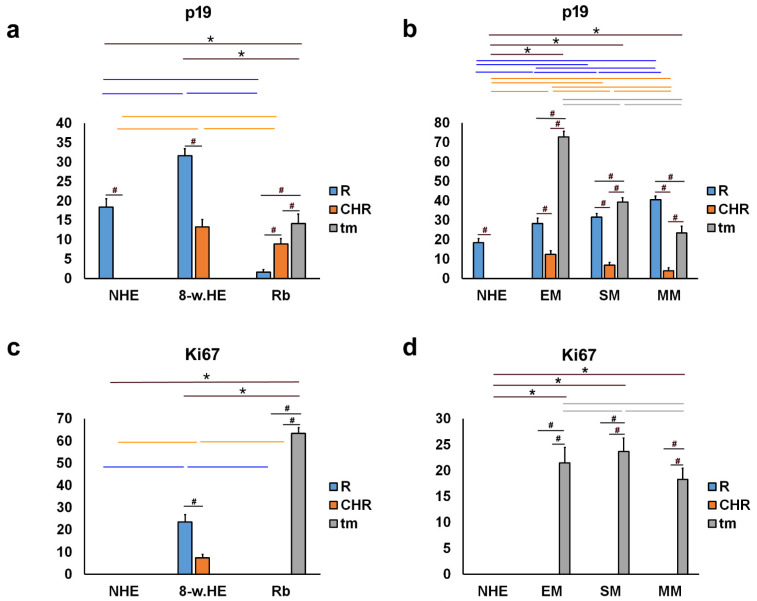
Quantitative analysis of p19 and Ki67 expression. Threshold area percentage of p19 and Ki67 in the normal human eye (NHE), the eye section of an 8-week human embryo (8-w.HE), retinoblastoma (Rb), epithelioid melanoma (EM), spindle melanoma (SM), and myxoid melanoma (MM). Data are shown as mean ± SD. #—statistically significant differences (comparison between retina, choroid and tumor in the same specimens); orange line—statistically significant differences-comparison of the choroid between NHE, 8-w.HE, tumor and different type of melanomas); blue line—statistically significant differences-comparison of the retina between NHE, 8-w.HE, tumor and different type of melanomas); grey line—statistically significant differences-comparison of the tumor tissue between different type of melanomas; *—statistically significant differences (comparison between Rb and retina or melanoma and choroid) (ANOVA and Tukey post-hoc test). All significant differences are presented in Supplementary Tables S1–S5.

**Figure 4:**
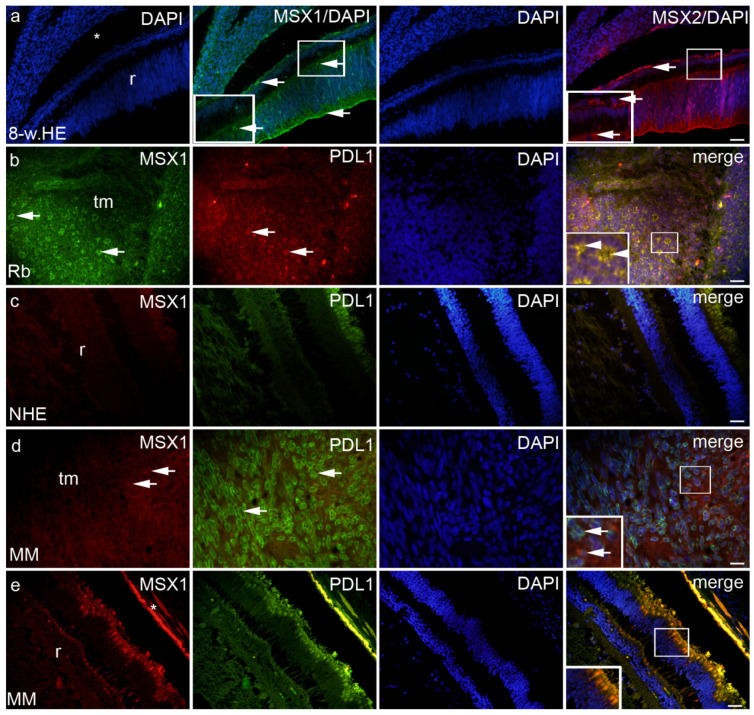
MSX1, MSX2 and PDL1 can be seen in the eye section of an 8-week human embryo–8-w.HE (**a**), retinoblastoma–Rb (**b**), normal human eye-NHE (**c**), myxoid melanoma-MM (**d**,**e**), inset on panel e—photoreceptors; r—retina, *—choroidea, tm—tumor tissue. Co-localization of MSX1 and PDL1 positive cells (arrowheads in the inset of magnification frame). Double immunofluorescence staining to MSX1 (first column), PDL1 (second column), DAPI (third column), MSX2 and merge (fourth column)—exceptions from this path are labelled on the panel. Magnification frames provide detail with positive cells (arrows) or co-localization (arrowheads). Scale bar for all panels is 25 μm.

**Figure 5:**
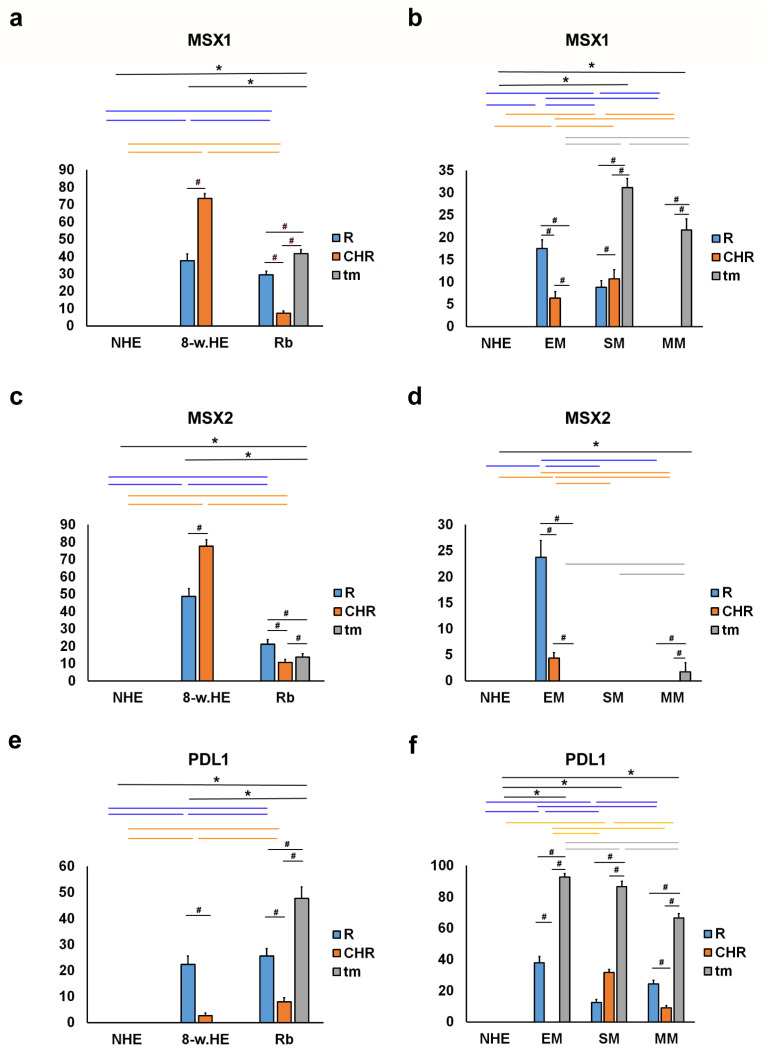
Quantitative analysis of MSX1, MSX2 and PDL1 expression. The threshold area percentage of MSX1, MSX2 and PDL1 in the normal human eye (NHE), the eye section of an 8-week human embryo (8-w.HE), retinoblastoma (Rb), epithelioid melanoma (EM), spindle melanoma (SM), and myxoid melanoma (MM). Data are shown as mean ± SD. #—statistically significant differences (comparison between retina, choroid and tumor in the same specimens); blue line—statistically significant differences-comparison of the retina between NHE, 8-w.HE, tumor and different type of melanomas); grey line—statistically significant differences-comparison of the tumor tissue between different type of melanomas; *—statistically significant differences (comparison between Rb and retina or melanoma and choroid) (ANOVA and Tukey post-hoc test). All significant differences are presented in Supplementary Tables S5–S10.

**Figure 6:**
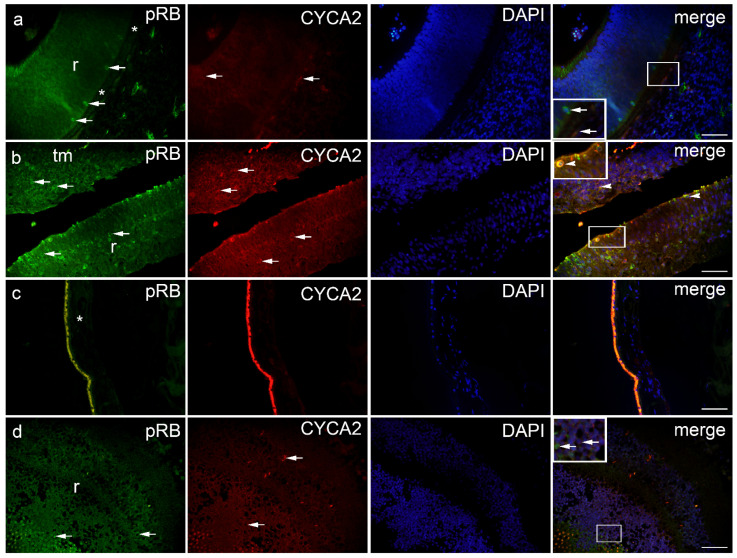
pRB and CYCA2 positive cells (arrows) can be seen in the eye section of an 8-week human embryo-8-w.HE (**a**), retinoblastoma-Rb (**b**), normal human eye-NHE (**c**) and epithelioid melanoma-EM (**d**); r—retina, *—choroidea, tm—tumor tissue. Co-localization of pRB and CYCA2 positive cells (arrowhead in the inset of magnification frame). Double immunofluorescence staining to pRB (first column), CYCA2 (second column), DAPI (third column), merge (fourth column). Magnification frames provide detail with positive cells (arrows) or co-localization (arrowheads). Scale bar 25 μm.

Expression patterns and quantitative and semiquantitative analysis of the markers p19, Ki-67, MSX1, MSX2, PDL1, pRB, and CYCLINA2 were performed on tissue sections from week 8 of human eye development, retinoblastoma, normal human eye, and ocular melanomas using hematoxylin and eosin (Figure 1) and immunofluorescence staining and quantitative analysis (Figure 2, Figure 3, Figure 4, Figure 5 and Figure 6). Semiquantitative expression of the above markers is shown in Table 1.

### 2.1. p19 and Ki67

p19-positive cells were observed in the eye section of an 8-week-old human embryo in the retina and choroid (Figure 2a), in retinoblastoma (unaffected retina, choroid, and tumor tissue) (Figure 2b), in the retina of the normal human eye (Figure 2c), and in various types of melanomas (retina, choroid, and tumor tissue) (Figure 2d). The expression of p19 was significantly lower in the retina of the normal human eye compared with the eye section of an 8-week-old human embryo. In the normal human eye and the eye section of an 8-week-old human embryo, the expression of p19 was significantly higher in the retina compared with the choroid. In contrast, the expression of p19 was lowest in retinoblastoma and significantly lower in the retina compared with the choroid and tumor tissue. However, compared with the retina of the normal human eye and the eye section of an 8-week-old human embryo, the expression of p19 was significantly lower in Rb tumor tissue (Figure 3a). In the different types of melanomas, the expression of p19 was also higher in the retina compared with the choroid. However, the expression of p19 was higher in the tumor tissue of the different melanoma types than in the choroid of the same eye and also than in the choroid of the normal human eye, where p19 was not expressed at all (Figure 3b).

Ki67-positive cells were observed in the eye section of an 8-week-old human embryo in both retina and choroid (Figure 2a), whereas they were positive only in tumor tissue in retinoblastoma (Figure 2b) and in various types of melanomas (Figure 2d). Ki67 expression was not observed in the retina or choroid of the normal human eye (Figure 2c), nor in retinoblastoma or melanoma (Figure 3c,d). However, we found expression of Ki67 in the retina and choroid of the eye of an 8-week-old human embryo, which was significantly higher in the retina than in the choroid. In addition, Ki67 expression was high in retinoblastoma tumor tissue (Figure 3c) and in all types of melanomas examined (Figure 3d).

p19- and Ki67-positive cells co-localised in the retina and choroid only in the eye of an 8-week-old human embryo.

### 2.2. MSX1, MSX2 and PDL1

MSX1-positive cells were observed in the eye section of an 8-week-old human embryo in the retina and choroid (Figure 4a), in retinoblastoma (retina, choroid, and tumor tissue) (Figure 4b), in spindle melanoma (retina, choroid, and tumor tissue), in myxoid only in tumor tissue (Figure 4d), and in epithelioid melanoma in the retina and choroid. No expression of MSX1 was found in the retina and choroid of the NHE (Figure 4c and Figure 5a,b). However, the expression of MSX1 was high in the eye of an 8-week-old human embryo, being significantly higher in the choroid compared with the retina (Figure 5a). In addition, retinoblastoma showed moderate expression of MSX1, which was highest in tumor tissue and lowest in the choroid (Figure 5a). MSX1 was also expressed in the retina and choroid of eyes with epithelioid and spindle-shaped melanomas but was not present in the retina or choroid of eyes with myxoid melanomas (Figure 5b). Interestingly, the expression of MSX1 differed markedly between the different melanoma types-it was absent in epithelioid melanoma, higher in myxoid melanoma, and highest in spindle melanoma.

MSX2-positive cells were observed in the eye section of an 8-week-old human embryo in the retina and choroid (Figure 4a) in retinoblastoma (remaining retina, choroid, and tumor tissue), in myxoid in tumor tissue only, and in epithelioid melanoma in the retina and choroid. Expression of MSX2 was not found in the retina or choroid of the NHE (Figure 4c and Figure 5c,d). However, similar to MSX1, expression of MSX2 was high in the eye of an 8-week-old human embryo, being significantly higher in the choroid than in the retina (Figure 5c). In addition, retinoblastoma showed moderate expression of MSX2, which was highest in the retina and lowest in the choroid (Figure 5c). MSX2 was also expressed in the retina and choroid of epithelioid melanoma, with significantly higher expression in the retina but not in the retina or choroid of spindle melanoma or myxoid melanoma (Figure 5d). Interestingly, the expression of MSX2 was low only in myxoid melanoma.

PDL1-positive cells were observed in the eye section of an 8-week-old human embryo in the retina and choroids, retinoblastomas (retina, choroids, and tumor tissue) (Figure 4b), and various types of melanomas (retina, choroids, and tumor tissue) (Figure 4d), with the exception of epithelioid melanoma, which had no PDL1 expression in the choroids. We found no PDL1 expression in the structures of the normal human eye (Figure 5e,f). In the eye of an 8-week-old human embryo, PDL1 expression was moderate in the cones and rods of the retina and low in the choroid, and it was similar in retinoblastoma. In addition, high expression of PDL1 was detected in the tumor tissue of retinoblastoma, which was significantly higher than the expression in the retina and choroid (Figure 5e). PDL1 was also expressed in the retina of epithelioid melanoma, spindle melanoma, and myxoid melanoma, whereas it was present in the choroid of spindle and myxoid melanoma (Figure 5f). As expected, the expression of PDL1 was highest in the tumor tissue of all three types of melanomas studied (EM, SM, and MM; Figure 5f).

### 2.3. pRB and CYCA2

**Figure 7:**
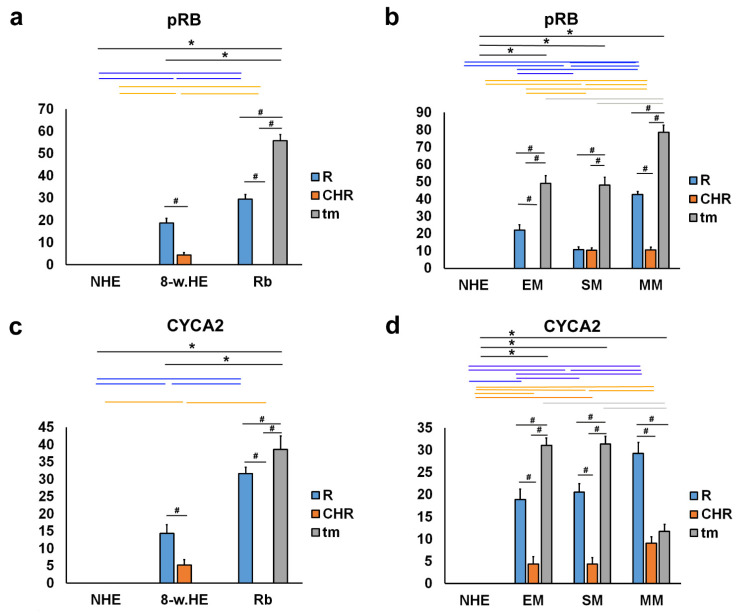
Quantitative analysis of pRB and CYC7A2 expression. The threshold area percentage of pRB and CYC7A2 in the normal human eye (NHE), the eye section of an 8-week human embryo (8-w.HE), retinoblastoma (Rb), epithelioid melanoma (EM), spindle melanoma (SM), and myxoid melanoma (MM). Data are shown as mean ± SD. #—statistically significant differences (comparison between retina, choroid and tumor of the same specimens); blue line—statistically significant differences-comparison of the retina between NHE, 8-w.HE, tumor and different type of melanomas); grey line—statistically significant differences-comparison of the tumor tissue between different type of melanomas; *—statistically significant differences (comparison between Rb and retina or melanoma and choroid) (ANOVA and Tukey post-hoc test). All significant differences are presented in Supplementary Tables S11–S14.

pRB-positive cells were observed in the eye section of an 8-week-old human embryo in the retina and choroids (Figure 6a), in retinoblastoma (retina and tumor tissue) (Figure 6b), and in various types of melanomas (retina, choroids, and tumor tissue) (Figure 6d), with the exception of epithelioid melanoma, which had no pRB expression in the choroids. Expression of pRB was not found in the retina and choroid of the normal human eye (Figure 7a,b). However, strong expression of pRB was found in the photoreceptors of the retina, whereas the choroid of an 8-week-old human embryo showed low expression. In retinoblastoma, expression of pRB was absent in the choroid, whereas it was high in tumor tissue and strong in the retina. The expression of pRB in retinoblastoma tumor tissue was significantly higher compared with the retina of the normal human eye and the eye of an 8-week-old human embryo (Figure 7a). In melanoma tumor tissue, the expression of pRB was highest in myxoid melanoma and significantly higher than in epithelioid and spindle melanoma tumor tissue. pRB expression also occurred in the retina of all three melanoma types and in the choroid of spindle-shaped and myxoid melanoma (Figure 7b).

CYCA2-positive cells were observed in the eye section of an 8-week-old human embryo in the retina and choroids (Figure 6a), in retinoblastoma (remaining retina and tumor tissue) (Figure 6b), and in various types of melanomas (retina, choroids, and tumor tissue) (Figure 6d). No CYCA2 expression was detected in normal human eye in the retina and choroid (Figure 7c,d). The change in the expression patterns of CYCA2 resembled those of pRB in the normal human eye, in an 8-week-old human embryo, and in retinoblastoma. Weak expression of CYCA2 was found in the retina, whereas low expression was detected in the choroid of an 8-week human embryo. In retinoblastoma, expression of pRB was not found in the choroid, whereas it was high in tumor tissue and retina. The expression of CYCA2 in the tumor tissue of retinoblastoma was significantly higher compared with the retina of the same eye; also compared with the retina of a normal human eye and an 8-week-old human embryo (Figure 7c). In all three types of melanomas studied, CYCA2 expression was found in tumor tissue, retina, and choroid. The highest CYCA2 expression was found in epithelioid and spindle-shaped melanoma tumor tissue, whereas it was lower in myxoid tumor tissue. Strong CYCA2 expression was also found in melanoma retina, being highest in myxoid melanoma (Figure 7d).

## 3. Discussion

In our study, we investigated the temporal and spatial expression pattern of cell cycle markers and proliferation factors during embryogenesis and tumorigenesis of the human eye. We compared the expression of these markers in tissue sections of the developing human eye at week 8, the normal human postnatal eye, and in retinoblastomas and choroidal melanomas.

The cell cycle factor ubiquitously expressed during organogenesis is p19, which regulates physiological withdrawal from the cell cycle and terminal differentiation of various progenitor cell populations. We found immunoreactive p19 cells in the retina and choroid of an 8-week-old human embryo, in the retina of a normal human eye, in an eye with retinoblastoma, and in various types of melanomas. However, the expression of p19 was lower in the postnatal retina compared with the embryonic retina. These results are consistent with the already known role of p19 in the cell cycle and with the high Ki-67 expression that we found in the embryonic human eye in this study. In our previous work (Matas et al.), we observed the expression of Ki-67 in the human eye in the late embryonic period (7th and 8th weeks of gestation). Ki-67 was used as a marker for the detection of proliferation in differentiating cells in various developing ocular compartments. However, the quantitative aspect of Ki-67 has not been investigated. Therefore, in this study, we investigated the quantitative change in Ki-67 expression during development and in ocular tumorigenesis. In our data, we found the same strong expression in both retina and choroid as in our previous work [19].

Co-localization of p19 and Ki67 in the same cells was found only occasionally in the retina and choroid of an 8-week-old human embryo. This is not surprising considering that previous studies have also shown expression of Ki67 in all phases of the cell cycle except the quiescent phase (G0) during human eye development [20]. Consistent with these data, the overlap of p19 expression with proliferation markers (Ki67, cyclin A2, and pRB) found in developing human tooth germs reinforces the relationship between p19 expression and proliferation activity during embryonic development [8]. The higher expression of p19 in the retina compared with the choroid is consistent with the marked proliferative activity of the retina during development. On the other hand, the low expression of p19 in retinoblastoma is consistent with the inhibitory role of p19 in the cell cycle. This may be one of the mechanisms for the high proliferative activity in retinoblastoma. The highest expression of p19 was found in tumor tissues from epithelioid melanomas. In contrast to the normal human eye, we found expression of p19 in the choroid of the eye in all types of melanomas examined. This may be related to the origin of choroidal melanoma from choroidal melanocytes. In addition to the normal embryonic human eye, we found high Ki67 expression in the tumor tissue of eyes with retinoblastoma and various types of choroidal melanoma. This is consistent with the known role of Ki-67 in cell proliferation processes [21].

The expression patterns of MSX1 and MSX2 were similar in 8-week human embryo and retinoblastoma, whereas both were absent in postnatal human eye. In choroidal melanoma, it is interesting to note that the expression patterns of MSX1 and MSX2 vary between tumor types. They were not found in the tumor tissue of epitheloid melanoma, but in this type of melanoma, high expression was found in unaffected remnants of choroidal and retinal tissue. In spindle and myxoid melanoma, on the other hand, MSX1 expression was high in tumor tissue. These drastic differences in the expression of MSX1 between epithelioid melanoma, on the one hand, and spindle and myxoid melanoma, on the other hand, may have prognostic significance for the poor prognosis of epithelioid melanoma [22]. Epithelial cells in epithelioid melanomas are more differentiated cell types of ectodermal origin, and the downregulation of MSX1 and MSX2 expression in this type of tumor may indicate a lack of tumor suppression. In contrast, the spindle type of melanoma is derived from fibroblasts, a less differentiated cell type with stronger MSX1 expression. In myxoid melanoma, the proportion of spindle cell type and its MSX1 expression likely indicates a better prognosis. In addition, myxoid melanoma may represent the epithelial-to-mesenchymal transition (EMT) [23]. MSX1 and MSX2 are known transcription factors for their role in EMT, which may suggest their involvement in malignant transformation of melanocytes [8].

PDL1 was expressed in the eye of an 8-week-old human embryo and in retinoblastomas and in several melanoma types except the epithelioid type, which had absence of PDL1 expression in choroid. It is surprising that PDL1 is strongly expressed in retinoblastoma, a low-immunogenic neoplasm [24]. However, expression of the PDL1 protein is an important link by which tumors evade the immune system in cancer. The mechanism of this response is the upregulation of the PDL1 protein, which leads to T cell exhaustion [21]. In addition, EMT helps to orchestrate immunosuppression and evade immune surveillance [25].

Transcription factor pRB is a negative regulator of cell proliferation [26]. However, the hyperphosphorylated form of pRB used in our study may be indicative of cell cycle progression and entry into mitosis. Indeed, we found pRB expression during human eye development as well as in retinoblastomas and various types of melanomas, whereas pRB expression was not found in the postnatal healthy human eye. In addition, several groups of cyclin-dependent kinases (Cdks) were found to be involved in the modulation of the pRB response [27]. In Ckds, CYCA2 is a critical regulator of the cell division cycle commonly associated with dividing cells. In our study, we observed the expression of CYCA2 in the developing human eye and retinoblastoma, similar to the expression of pRB. Different types of melanomas showed high expression of CYCA2 in tumor tissue, whereas myxoid melanoma showed higher expression in the retina compared with the tumor part. No CYCA2 expression was detected in the retina and choroid of the normal human eye. Kuźbicki et al. demonstrated that melanoma development and progression are associated with changes in CDK-2 expression and that this may have prognostic significance [28].

The lack of our study is omitting some of very important gene expressions in eye development and tumorigenesis, such as p21, p27, OTX2, SOX2/3, PAX6, RAX1, and cy-clin E. Loss of expression or function of p21 (CDKN1A) and p27 (CDKN1B), the two CDK inhibitors of the G1 checkpoint, is associated with the development or progression of many human malignancies [29]. Loss of OTX2 expression may play a role in retinoblastoma development by contributing to the increase in phosphorylation of RB and its effects [30]. SOX2 gene expression in RB tissues increases with progression of clinical stage [31], while the role of SOX2 in melanoma is controversial [32]. Loss of RAX1 expression in eye development results in eyeless mice [33]. Cyclin E activation in late G1 phase is critical for pRb inactivation and G1-S phase cell cycle progression [34]. Therefore, investigation of these additional markers in further studies would allow a more systematic approach.

In conclusion, the observed expression differences of the analyzed markers correlate with the origin and stage of cell differentiation of the tissue samples. The fine balance of expression could play a role in both human eye development and ocular tumorigenesis. Therefore, understanding their precise expression patterns could open new avenues for potential therapeutic targeting and better understanding of the mechanisms underlying ocular developmental plasticity and neoplasm formation.

## 4. Materials and Methods

### 4.1. Tissue Procurement

Embryonic tissues were obtained from the archives of the Department of Anatomy, Histology and Embryology, Faculty of Medicine, University of Split. We used 5 conceptuses at the 8th week of development according to the Carnegie staging system based on morphology as we have described previously [35,36,37]. Samples of normal human eye (five samples), retinoblastoma (five samples), and melanoma (15 samples) were collected at the Department of Pathology, Split University Hospital. The study was performed with the approval of the Ethics Committee of the University Hospital of Split in accordance with the Declaration of Helsinki and its updates.

All specimens collected were evaluated by a pathologist who classified the diagnoses. Retinoblastoma samples were tested for RB1 mutation, while melanoma samples were tested for BRAFV600E, BRCA2, CDK4, CDKN2A, PTEN, and p53. All samples used did not have any of the tested mutations. Only well-preserved tissue was used after external examination; macerated or poorly preserved material was discarded. Standard hematoxylin and eosin staining was performed on each tissue block to confirm proper preservation of the material.

### 4.2. Double Immunofluorescence

Tissue samples were fixed in 4% paraformaldehyde dissolved in phosphate buffered saline (PBS), dehydrated in graded ethanol and embedded in kerosene. Serial sections 5 µm thick were cut in a transverse plane and mounted on glass slides as previously described [38,39]. After deparaffinization of the slides and rinsing in PBS, the sections were incubated overnight with an appropriate combination of primary antibodies in a humidity chamber at 21 °C (StainTray slide staining system; Sigma-Aldrich, St. Louis, MO, USA). Protein Block (ab64226; Abcam, Cambridge, UK) was applied before the application of the primary antibodies to avoid nonspecific staining. The primary and secondary antibodies used are listed in Table 2. After incubation with the primary antibodies, the sections were rinsed in PBS and incubated with an appropriate combination of secondary antibodies for one hour. 4′,6-Diamidino-2-phenylindole dihydrochloride (DAPI) was used to stain the nuclei. After final rinsing in PBS, sections were mounted with medium, coverslipped, and analyzed using an Olympus BX51 microscope (Olympus, Tokyo, Japan).

### 4.3. Data Analysis

Tissue section analysis was performed on images taken with a DP71 digital camera (Olympus, Tokyo, Japan) at high magnification (×400) and mounted on a fluorescence microscope (Olympus BX51, Tokyo, Japan). Only the retina, choroid, and tumor tissue area of retinoblastoma and melanoma were of interest. Images were analyzed using ImageJ software (Rasband, W.S., ImageJ, U. S. National Institutes of Health, Bethesda, MD, USA, https://imagej.nih.gov/ij/, (accessed on 13 April 2022), 1997–2018.) and Adobe Photoshop (San Jose, CA, USA). We measured the percent threshold on at least 25 structures (retina, choroid, and tumor tissue) per group. The surface of positive cells was labeled, and their percentage was measured in the squares (20 × 20 μm). The percentage threshold (percentage of surface covered with positive cells) was analyzed in five randomly selected squares of each image and compared between the different regions (retina, choroid, and tumor tissue). Four independent investigators analyzed the data.

### 4.4. Semiquantitative Analysis

The intensity of tissue staining with the primary antibodies used was described semiquantitatively by four categories of staining intensity, with (−) indicating the absence of any reactivity, (+) indicating mild reactivity, (++) indicating moderate reactivity, and (+++) indicating strong reactivity (Table 1). Four investigators independently analyzed staining intensity in retina, choroid, and tumor tissue of retinoblastoma and melanoma (Table 1).

### 4.5. Statistical Analysis

For the statistical analysis between the groups and structures, one-way ANOVA was followed by Tukey post hoc test performed using GraphPad Prism software (La Jolla, California, USA, www.graphpad.com). In each region of interest (20 × 20 μm), threshold area per percent was calculated and expressed as mean ± standard deviation (SD), with statistical significance set at *p* < 0.05.

## Figures and Tables

**Table 1:** Immunoreactivity to specific antibodies compared: 8w human eye development, retinoblastoma, normal human eye, choroidal melanomas in three subtypes, epiteloid, spindle, and myxoid.

Antibodies								
Structure		p19	Ki67	Msx1	Msx2	PD-L1	pRB	cyclA2
**8w human eye**	retina	**+++**	**+++**	**++**	**+++**	++ * cr	+++ * ph	**+**
	choroid	**++**	**+++**	**++**	**+++**	**−**	**−**	**+**
**Retinoblastoma**	retina	**++**	**−**	**+++**	**++**	**+++**	+++ * n	**+++**
	choroid	**++**	**−**	**+++**	**++**	**+++**	**−**	**−**
	tm	**+**	**+++**	**++**	**+**	**++**	+++ * c	**+**
**Normal human eye**	retina	**+**	**−**	**−**	**−**	**−**	**−**	**−**
	choroid	**−**	**−**	**−**	**−**	**−**	**−**	**−**
**Melanoma** **epiteloid** **type**	retina	**+++**	**−**	**++**	**++**	**+++**	**+++**	**+++**
	choroid	**++**	**−**	**++**	**−**	**−**	**−**	**+**
	tm	**+++**	**+++**	**−**	**−**	**+++**	**+++**	**+**
**Melanoma** **spindle** **type**	retina	**+**	**−**	**+**	**−**	**+++**	**+++**	**+**
	choroid	**+**	**−**	**+**	**−**	**+++**	**+++**	**+**
	tm	**+**	**+++**	**++**	**−**	**+++**	**+++**	**++**
**Melanoma** **myxoid** **type**	retina	**+**	**−**	++ * ph	**−**	**+++**	**++**	**+++**
	choroid	**+**	**−**	**−**	**−**	**+++**	++ * c	**++/+++**
	tm	**+++**	**+++**	**+**	**+**	**+++**	**+++**	**+**

+++ strong reactivity; ++ moderate reactivity; + mild reactivity; − no reactivity; w—week of development, *—exception, ph—photoreceptors, cr—cones and rods, n—nucleus, c—cytoplasm.

**Table 2:** Primary and secondary antibodies used in the study.

Antibodies		Host	Dilution	Source
Primary	p19 INK4d	Rabbit	1:300	ab102842 Abcam (Cambridge, UK)
	[EPR182(N)] to phospho S780	Rabbit	1:300	ab173289 Abcam (Cambridge, UK)
	Ki67	Mouse	1:100	M7240, DAKO, Glostrup, Denmark
	MSX1	Goat	1:300	ab93287 Abcam (Cambridge, UK)
	Cyclin A2 (E23.1)	Mouse	1:300	ab38 Abcam (Cambridge, UK)
	[ABM4E54] to PDL1	Mouse	1:500	ab210931 Abcam (Cambridge, UK)
	Msx2/Hox8-N-terminal	Rabbit	1:300	ab190070 Abcam (Cambridge, UK)
Secondary	Anti-Rabbit IgG H&L (Alexa Fluor 488)	Donkey	1:500	ab150073 Abcam (Cambridge, UK)
	Anti-Mouse Rhodamin	Goat	1:300	AP124R Jackson Immuno Research Laboratories, Inc., (Baltimore, PA, USA)
	Anti-Rabbit IgG H&L (Alexa Fluor 594)	Donkey	1:500	ab150076 Abcam (Cambridge, UK)
	Anti-Goat IgG H&L (Alexa Fluor^®^ 594)	Donkey	1:500	ab150132 Abcam (Cambridge, UK)
	Anti-Mouse IgG H&L (Alexa Fluor 488)	Donkey	1:500	ab150105 Abcam (Cambridge, UK)

## Data Availability

The study did not report any data.

## Supplementary Materials

The following supporting information can be downloaded at: https://www.mdpi.com/article/10.3390/ijms23169421/s1.
