# Supplementary material for: Expression of Cell Cycle Markers and Proliferation Factors during Human Eye Embryogenesis and Tumorigenesis
Source: Int J Mol Sci. 2022 Aug 20;23(16):9421. doi: 10.3390/ijms23169421 (PMC9409163; doi:10.3390/ijms23169421)
Supplement: Supplementary file 1 [file ijms-23-09421-s001.zip › ijms-1777094-supplementary.pdf]

Supplementary table S1. Expression of p19 - comparison between structures in normal human eye (NHE), the eye section of an 8-week human embryo (8-w.HE) and retinoblastoma (Rb). Tukey's multiple comparisons test P-values.

		NHE		8-w.HE		rRb		
		R	CHR	R	CHR	R	CHR	T
NHE	R		<0.0001	<0.0001		<0.0001		<0.0001
	CHR				<0.0001		<0.0001	
8-w.HE	R				<0.0001	<0.0001		<0.0001
	CHR						<0.0001	
rRb	R						<0.0001	<0.0001
	CHR							<0.0001
	T							

Supplementary table S2. Expression of p19 - comparison between structures in normal human eye (NHE), epithelioid melanoma (EM), spindle melanoma (SM), and myxoid melanoma (MM). Tukey's multiple comparisons test P-values.

		EM			SM			MM		
		R	CHR	T	R	CHR	T	R	CHR	T
NHE	R	<0.0001			<0.0001			<0.0001		
	CHR		<0.0001	<0.0001		<0.0001	<0.0001		<0.0001	<0.0001
EM	R		<0.0001	<0.0001	<0.0001			<0.0001		
	CHR			<0.0001		<0.0001			<0.0001	
	T						<0.0001			<0.0001
SM	R					<0.0001	<0.0001	<0.0001		
	CHR						<0.0001		0.0002	
	T									<0.0001
MM	R								<0.0001	<0.0001
	CHR									<0.0001
	T									

Supplementary table S3. Expression of Ki67 - comparison between structures in normal human eye (NHE), the eye section of an 8-week human embryo (8-w.HE) and retinoblastoma (Rb). Tukey's multiple comparisons test P-values.

		NHE		8-w.HE		rRb		
		R	CHR	R	CHR	R	CHR	T
NHE	R		ns	<0.0001		ns		<0.0001
	CHR				<0.0001		ns	
8-w.HE	R				<0.0001	<0.0001		<0.0001
	CHR						<0.0001	
rRb	R						ns	<0.0001
	CHR							<0.0001
	T							

Supplementary table S4. Expression of Ki67 - comparison between structures in normal human eye (NHE), epithelioid melanoma (EM), spindle melanoma (SM), and myxoid melanoma (MM). Tukey's multiple comparisons test P-values.

		EM			SM			MM		
		R	CHR	T	R	CHR	T	R	CHR	T
NHE	R	ns			ns			ns		
	CHR		ns	<0.0001		ns	<0.0001		ns	<0.0001
EM	R		ns	<0.0001	<0.0001			<0.0001		
	CHR			<0.0001		<0.0001			<0.0001	
	T						<0.0001			<0.0001
SM	R					ns	<0.0001	<0.0001		
	CHR						<0.0001		ns	
	T									<0.0001
MM	R								ns	<0.0001
	CHR									<0.0001
	T									

Supplementary table S5. Expression of MSX1 - comparison between structures in normal human eye (NHE), the eye section of an 8-week human embryo (8-w.HE) and retinoblastoma (Rb). Tukey's multiple comparisons test P-values.

		NHE		8-w.HE		rRb		
		R	CHR	R	CHR	R	CHR	T
NHE	R		ns	<0.0001		<0.0001		<0.0001
	CHR				<0.0001		<0.0001	
8-w.HE	R				<0.0001	<0.0001		<0.0001
	CHR						<0.0001	
rRb	R						<0.0001	<0.0001
	CHR							<0.0001
	T							

Supplementary table S6. Expression of MSX1 - comparison between structures in normal human eye (NHE), epithelioid melanoma (EM), spindle melanoma (SM), and myxoid melanoma (MM). Tukey's multiple comparisons test P-values.

		EM			SM			MM		
		R	CHR	T	R	CHR	T	R	CHR	T
NHE	R	<0.0001			<0.0001			ns		
	CHR		<0.0001	ns		<0.0001	<0.0001		ns	<0.0001
EM	R		<0.0001	<0.0001	<0.0001			<0.0001		
	CHR			<0.0001		<0.0001			<0.0001	
	T						<0.0001			<0.0001

SM	R					0.0003	<0.0001	<0.0001		
	CHR						<0.0001		<0.0001	
	T									<0.0001
MM	R								ns	<0.0001
	CHR									<0.0001
	T									

Supplementary table S7. Expression of MSX2 - comparison between structures in normal human eye (NHE), the eye section of an 8-week human embryo (8-w.HE) and retinoblastoma (Rb). Tukey's multiple comparisons test P-values.

		NHE		8-w.HE		rRb		
		R	CHR	R	CHR	R	CHR	T
NHE	R		ns	<0.0001		<0.0001		<0.0001
	CHR				<0.0001		<0.0001	
8-w.HE	R				<0.0001	<0.0001		<0.0001
	CHR						<0.0001	
rRb	R						<0.0001	<0.0001
	CHR							0.0006
	T							

Supplementary table S8. Expression of MSX2 - comparison between structures in normal human eye (NHE), epithelioid melanoma (EM), spindle melanoma (SM), and myxoid melanoma (MM). Tukey's multiple comparisons test P-values.

		EM			SM			MM		
		R	CHR	T	R	CHR	T	R	CHR	T
NHE	R	<0.0001			ns			ns		
	CHR		<0.0001	ns		ns	ns		ns	<0.0001
EM	R		<0.0001	<0.0001	<0.0001			<0.0001		
	CHR			<0.0001		<0.0001			<0.0001	
	T						ns			<0.0001
SM	R					ns	ns	ns		
	CHR						ns		ns	
	T									<0.0001
MM	R								ns	<0.0001
	CHR									<0.0001
	T									

Supplementary table S9. Expression of PDL1 - comparison between structures in normal human eye (NHE), the eye section of an 8-week human embryo (8-w.HE) and retinoblastoma (Rb). Tukey's multiple comparisons test P-values.

		NHE		8-w.HE		rRb		
		R	CHR	R	CHR	R	CHR	T
NHE	R		ns	<0.0001		<0.0001		<0.0001

	CHR				0.0040		<0.0001	
8-w.HE	R				<0.0001	0.001		<0.0001
	CHR						<0.0001	
rRb	R						<0.0001	<0.0001
	CHR							<0.0001
	T							

Supplementary table S10. Expression of PDL1 - comparison between structures in normal human eye (NHE), epithelioid melanoma (EM), spindle melanoma (SM), and myxoid melanoma (MM). Tukey's multiple comparisons test P-values.

		EM			SM			MM		
		R	CHR	T	R	CHR	T	R	CHR	T
NHE	R	<0.0001			<0.0001			<0.0001		
	CHR		ns	<0.0001		<0.0001	<0.0001		<0.0001	<0.0001
EM	R		<0.0001	<0.0001	<0.0001			<0.0001		
	CHR			<0.0001		<0.0001			<0.0001	
	T						<0.0001			<0.0001
SM	R					<0.0001	<0.0001	<0.0001		
	CHR						<0.0001		<0.0001	
	T									<0.0001
MM	R								<0.0001	<0.0001
	CHR									<0.0001
	T									

Supplementary table S11. Expression of pRB - comparison between structures in normal human eye (NHE), the eye section of an 8-week human embryo (8-w.HE) and retinoblastoma (Rb). Tukey's multiple comparisons test P-values.

		NHE		8-w.HE		rRb		
		R	CHR	R	CHR	R	CHR	T
NHE	R		ns	<0.0001		<0.0001		<0.0001
	CHR				<0.0001		ns	
8-w.HE	R				<0.0001	<0.0001		<0.0001
	CHR						<0.0001	
rRb	R						<0.0001	<0.0001
	CHR							<0.0001
	T							

Supplementary table S12. Expression of pRB - comparison between structures in normal human eye (NHE), epithelioid melanoma (EM), spindle melanoma (SM), and myxoid melanoma (MM). Tukey's multiple comparisons test P-values.

	EM	SM	MM

		R	CHR	T	R	CHR	T	R	CHR	T
NHE	R	<0.0001			<0.0001			<0.0001		
	CHR		ns	<0.0001		<0.0001	<0.0001		<0.0001	<0.0001
EM	R		<0.0001	<0.0001	<0.0001			<0.0001		
	CHR			<0.0001		<0.0001			<0.0001	
	T						ns			<0.0001
SM	R					ns	<0.0001	<0.0001		
	CHR						<0.0001		ns	
	T									<0.0001
MM	R								<0.0001	<0.0001
	CHR									<0.0001
	T									

Supplementary table S13. Expression of CYCA2 - comparison between structures in normal human eye (NHE), the eye section of an 8-week human embryo (8-w.HE) and retinoblastoma (Rb). Tukey's multiple comparisons test P-values.

		NHE		8-w.HE		rRb		
		R	CHR	R	CHR	R	CHR	T
NHE	R		ns	<0.0001		<0.0001		<0.0001
	CHR				<0.0001		ns	
8-w.HE	R				<0.0001	<0.0001		<0.0001
	CHR						<0.0001	
rRb	R						<0.0001	<0.0001
	CHR							<0.0001
	T							

Supplementary table S14. Expression of CYCA2 - comparison between structures in normal human eye (NHE), epithelioid melanoma (EM), spindle melanoma (SM), and myxoid melanoma (MM). Tukey's multiple comparisons test P-values.

		EM			SM			MM		
		R	CHR	T	R	CHR	T	R	CHR	T
NHE	R	<0.0001			<0.0001			<0.0001		
	CHR		<0.0001	<0.0001		<0.0001	<0.0001		<0.0001	<0.0001
EM	R		<0.0001	<0.0001	0.0264			<0.0001		
	CHR			<0.0001		ns			<0.0001	
	T						ns			<0.0001
SM	R					<0.0001	<0.0001	<0.0001		
	CHR						<0.0001		<0.0001	
	T									<0.0001
MM	R								<0.0001	<0.0001
	CHR									<0.0001
	T									
